# Screening and characterization of proline dehydrogenase flavoenzyme producing *Pseudomonas entomophila*

**Published:** 2011-12

**Authors:** H Shahbaz- Mohammadi, E Omidinia

**Affiliations:** Department of Biochemistry, Pasteur Institute of Iran, Pasteur Street, Tehran, Iran

**Keywords:** Characterization, Purification, Proline dehydrogenase (ProDH), *Pseudomonas entomophila*

## Abstract

**Background and Objectives:**

Proline dehydrogenase (ProDH; 1.5.99.8) plays an important role in specific determination of plasma proline level in biosensor and diagnostic kits. The goal of this research was to isolate and characterize ProDH enzyme from Iranian soil microorganisms.

**Materials and Methods:**

Screening of L-proline degradative enzymes from soil samples was carried out employing enrichment culture techniques. The isolate was characterized by biochemical reactions and specific PCR amplification. The target ProDH was purified and the effects of pH and temperature on the activity and stability were also tested.

**Results:**

Among the 250 isolates recovered from 40 soil samples, only one strain characterized as *Pseudomonas entomophila* displayed the highest enzyme activity toward L-proline (350 U/l) than others. The enzyme of interest was identified as a ProDH and had *K*_m_ value of 32 mM for L-proline. ProDH exhibited its best activity at temperature range of 25 to 35°C and its highest activity was achieved at 30°C. It was almost stable at temperatures between 25-30°C for 2 hours. The optimum pH activity of ProDH reaction was 8.5 and its activity was stable in pH range of 8.0-9.0 upto 24 hours. The enzyme was purified with a yield of 8.5% and a purification factor of 37.7. The molecular mass of the purified ProDH was about 40 kDa, and determined to be a monomeric protein.

**Conclusion:**

This is the first report concerning the ProDH production by a *P. entomophila* bacterium isolated from soil sample.

## INTRODUCTION

Enzymes have been used as potential biocatalysts with a wide range of applications in various industrial processes. They are responsible for all chemical functions in living organisms. Therefore, all living systems including animal, plant and microorganisms are sources of enzymes. However, for commercial applications of industrial enzymes, microbes are the major source. Currently, more than 90% of commercially available enzymes are produced by microorganisms (1, 2). Microorganisms are preferred to plants and animals as enzyme sources because of their rich diversity in nature, stability in different environments, lower production costs, higher enzyme contents and their potential use in biotechnology industry. In this area, isolation and screening of microorganisms from naturally occurring habitats to provide enzymes with novel properties is very useful (3). One of the interesting microbial enzymes is proline dehydrogenase (Pro DH; 1.5.99.8).

The metabolic pathway of proline includes conversion of proline to Δ1-pyrroline-5-carboxylate (P5C), followed by conversion to ornithine, metabolism in the urea cycle or to glutamate and introduction into the TCA cycle. The degradation of proline to the glutamate proceeds in two oxidative step by the actions of ProDH and Δ^1^-pyrroline-5-carboxylate dehydrogenase (P5CDH; P5C: NAD^+^ oxidoreductase, 1.5.1.12) enzymes. At first, ProDH catalyzes the transfer of two electrons from proline to a flavin adenine dinucleotide (FAD) cofactor to generate P5C and FADH_2_. Electrons from the reduced of the noncovalently associated FAD are subsequently transmitted to an acceptor in the respiratory chain (4). The human homolog of the ProDH plays critical roles in cancer prevention and schizophrenia (5). This enzyme has also potential utilization for specific determination of plasma proline level in biosensor and diagnostic kits (6). In the second step of proline oxidation, P5C is non-enzymatically hydrolyzed to glutamate- γ-semialdehyde (GSA), which is then oxidized into glutamate by P5CDH in a NAD^+^-dependent reaction. Inherited disorders in proline metabolism cause hyperprolinema diseases in humans. Deficiency of the ProDH and P5CDH activity results in type I and type II hyperprolinemia, respectively (7). Studying the proline metabolizing enzymes from bacteria provides valuable information on the structure and function of human version (8, 9). Furthermore, the bacterial ProDHs are potentially attractive for specific determination of plasma proline in diagnostic kits and proline biosensors (5, 6). Up to now, there have been many studies on screening of proline catabolic enzymes from different microbial sources such as *Escherichia coli* (7), *Pseudomonas aeruginosa* (10), *Salmonella typhimurium* (4) and *Bradyhizobium japonicum* (11). In this communication, we report the purification and characterization of ProDH from a newly isolated *P. entomophila*.

## MATERIALS AND METHODS

**Isolation and screening for proline degradative enzymes.** Soil samples were collected from different locations of Tehran during fall and winter seasons of 2006. The samples were placed in sterile polyethylene bags and maintained at 4° C until processed. One gram of each sample was suspended in PYP selective liquid medium that contained (per liter): 0.5% L-proline, 1 g NaCl, 2 g K_2_HPO_4_, 0.5 g MgSO_4_ .7H_2_O, 5 g yeast extract, 5 g polypeptone in 1 liter of tap water, pH 7.0, then incubated with shaking at 140 rpm for 48 h. Serial dilutions up to 10^-4^ were prepared. From each dilution, 0.05 ml was taken and spread on agar plates and incubated at 37°C for 24-36 h until the isolates formed colonies. The single uniform colonies were streaked at least three times to ensure purity (12). In secondary screening procedure, the ability to degrade L-proline was determined by thin layer chromatography (TLC). An aliquot (2 µl) of the culture medium was applied to silica gel TLC plates (silica gel 60F254 plate, 20×20 cm; Merck).The degradation of proline was identified by developing TLC plate in the solvent mixture of water: ethanol (3:7 by vol). The plate was then dried and visualized by spraying ninhydrin (0.2% [wt/vol] in acetone) followed by heating (13).

**Strain identification.** Identification of bacterium was performed by morphological characterization, biochemical methods and specific PCR amplification. Classification as Gram negative or Gram positive was done by Gram stain reaction and KOH test. Morphological characteristics of the isolated bacterium were also performed according to the standard method (14). Biochemical methods were conducted using well-established biochemical tests described in Bergey's Manual of Determinative Bacteriology (15). For the preparation of total genomic DNA, bacterial strain was first cultured as described in an earlier section. Chromosomal DNA from bacterial cell was purified according to the Doi protocol (16). The resultant DNA sample was suspended in a buffer containing 10 mM Tris-HCl (pH 7.6), 1 mM EDTA and 10 mM NaCl and stored at −20°C for further work. Complete 16S rRNA gene sequence was amplified from the chromosomal DNA of the isolated strain with primers 16SF (5’-AGAGTTTGATCCTGGCTCAG-'3) and 16SR (5’-CTACGGCTACCTTGTTACGA-'3) (17). PCR amplification was performed in volumes of 25 µl containing 20 pmol of each primer, 1X PCR buffer, 0.2 mM of each dNTP, 1.5 mM MgCl_2_, 0.3 µg template DNA and 2.5 units of *Taq* DNA polymerase. Nucleases free water was used to bring the reaction volume to 25 µl. The resultant PCR product was then analyzed in a 1.5% (w/v) horizontal agrose gel, excised from the gel and purified. The amplified 16S rDNA was cloned into pJET1.2 Vector (MBI Fermentas, St. Leon-Rot, Germany) and transformed into *E. coli* JM 107. Plasmid DNA vector was isolated from the positive clones using plasmid extraction protocol (18). Sequencing was performed by the commercial services of MacroGen Co. Ltd. (Seoul, Korea) with the appropriate sequencing primers. The 16S rDNA sequence of the isolate was aligned with the reference 16S rDNA sequences using the Basic Local Alignment Search Tool (BLAST) algorithm available in NCBI. Multiple alignment of sequences and calculations of levels of sequence similarity were performed by using ClustalW. Phylogenic trees were constructed via the neighbor-Joining (NJ) algorithm using the Molecular Evolution Genetic Analysis (MEGA) program, version 4.0 (19). Bootstrap test was done 1000 times to confirm the reliability and validity of inferred trees.

**Characterization of *P. entomophila* ProdH.** Purification of ProDH was performed by the modifications of methods previously described (4, 10, 21). The cells were harvested in the late exponential phase by centrifugation at 5000×g for 30 min at 4°C and washed twice with 0.9% NaCl solution. The washed cells (wet weight, about 20 g) were suspended in 50 ml of buffer A (100 mM Tris-HCl, pH 8.0) and disrupted by ultrasonic oscillator for 20 min. Cells and insoluble materials were removed by centrifugation at 4000 × g for 1 h at 4°C. The supernatant solution was used as the crude extract. A small aliquot of the supernatant containing the solubilized ProDH was used for enzyme activity assay experiments and its remainder was also precipitated with ammonium sulfate as described in next step. The enzyme solution was brought to 50% saturation by addition of solid ammonium sulfate solution under gentle stirring at 4°C. The supernatant was then centrifuged for 30 min at 5000 rpm to collect the precipitate. The pellet was redissolved in 20 ml of buffer B (70 mM Tris-HCl, pH 8.2 containing 0.5% (w/v) Tween-20 and 10% (v/v) glycerol) and dialyzed overnight at 4°C against at the same buffer. The dialyzed fraction was concentrated by ultrafiltration and subjected to a DEAE-Toyopearl column (diameter, 3 cm; length, 15 cm) equilibrated with buffer B using a fast performance liquid chromatography (FPLC) system (Sykam). The column was washed first with buffer B and subsequently washed with buffer B containing 50 mM KCl. ProDH was eluted with a stepwise linear salt gradient of KCl concentration (50- 150 mM) in buffer B at a flow rate of 3 ml/min. Fractions of 3 ml per tube were collected. The active enzyme fractions were pooled, concentrated, and stored at 4°C for various experiments. The concentrated enzyme was loaded on a Sephadex G-200 gel filtration (2.5 cm×120 cm), which had been equilibrated with buffer A. The flow rate was maintained at 1 ml/min. Fractions of 3 ml each was collected and absorbance at 280 nm was recorded. Purification process was analyzed by sodium dodecyl sulfate polyacrylamide gel electrophoresis (SDS-PAGE). SDS-PAGE was performed using discontinuous gels (10 cm×10 cm) with a 6% stacking and a 12% separating gel. The protein samples were boiled for 5 min in 10 mM Tris-HCl buffer (pH 7.0) containing 1% SDS, 80 mM 2-mercatoethonal and 15% glycerol. Electrophoresis was run at 30v and 10mA for 5h. Protein bands were visualized by staining with 0.025 Coomassie brilliant Blue R-250 in the mixture of 50% methanol and 10% acetate (18).

The effect of temperature on the enzymatic reaction of ProDH was analyzed by performing the enzyme assay at various temperatures (30–70°C). Reaction mixture was pre-incubated at the desired temperatures for 4 min. The thermal stability of ProDH was examined by incubating at different temperatures ranging from 30 to 70°C for 60 min and then cooling on ice-cold water. Residual activity was measured at every 10 min interval under standard assay conditions. The non-heated enzyme was used as a control (23). The effect of pH on the enzymatic reaction was evaluated by measuring the activity in the following buffer systems: 0.1 M sodium acetate (pH 3.0-5.0), 0.1 M potassium phosphate (pH 6.0–7.5), 0.1 M Tris-HCl (pH 8.0–9.0), 0.1 M glycine-NaOH (pH 9.0–11.0) and 0.1 M sodium carbonate (pH 11.5–12.0). To study the influence of pH on the stability of ProDH, crude enzyme was mixed with the above mentioned buffers at a ratio of 1:1 and then incubated at 4 °C for 24 h. Aliquots was taken at time intervals of 4 h and the residual activity was calculated under standard assay conditions. All experiments were done triplicate and repeated at least twice (23).

**Determination of enzyme activity and protein concentration.** ProDH activity was measured using the proline: INT (2-(p-iodophenyl)-3-(p-nitrophenyl)- 5-phenyltetrazolium chloride) oxidoreductase assay which was performed by INT as a terminal electron acceptor and phenazine methosulfate (PMS) as a mediator electron carrier. The standard reaction mixture was composed of 200 mM Tris-HCl, pH 8.5,200 mM L-proline, 0.2 mM FAD, 0.4 mM INT, 0.08 mM PMS and the enzyme in a total volume of 1ml. The increase in absorbance at 490 nm was estimated and corrected for blank values lacking proline. One unit (U) of ProDH activity was defined as the quantity of enzyme, which transfers electrons from 1 µmol of proline to INT per minute at 25°C (20). All assay experiments were done in triplicate and the average results were used for data analysis. The total protein concentration was determined by the method of Bradford using bovine serum albumin (BSA) as a standard (22).

## RESULTS

**Fig. 1:**
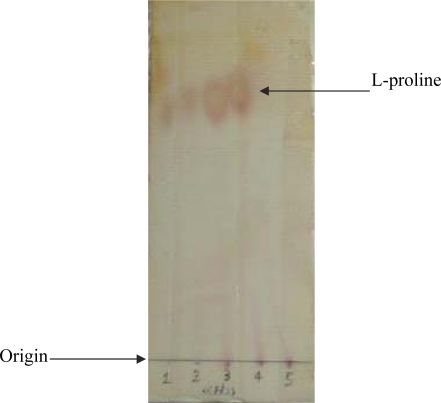
TLC profile of L-proline broth cultured with soil samples.

**Screening for L-proline metabolizing enzymes.** During the primary screening, 250 strains were isolated. Among them, 40 isolates, which participated in proline degradation, were identified. The spot of L-proline as indicating by an arrow in Fig. 1 was considered as inability of L-proline degradation. To confirm these selections, bacterial isolates, which were capable to utilize L-proline were cultivated in the production medium, and the enzyme activity was measured. From these 40 isolates, only strain POS-F84 which was isolated from the river clay, was found to be capable to metabolize proline (activity, 350 U/l) and selected for further studies.

**Fig. 2:**
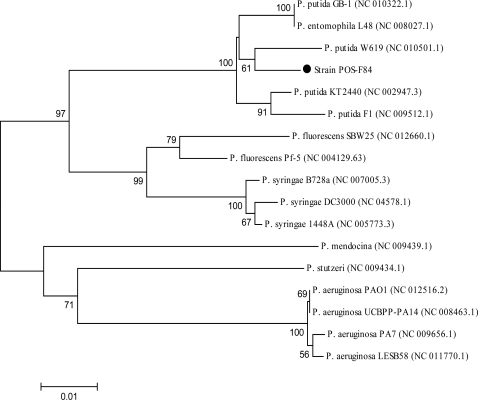
Phylogenetic analysis of the 16S rDNA sequences of POS-F84, and closely related members in the genus *Pseudomonas*. Numbers at nodes are levels of bootstrap support (%) based on Neighbor-joining (NJ) method of 1,000 resampled datasets. The bootstrap value below 50% is not shown. The scale bar indicates 0.01 nucleotide substitution per position. Isolate is marked.

**Fig. 3:**
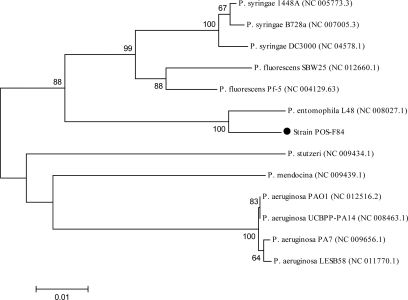
Phylogenetic tree based on ITS sequences of the isolated POS-F84 and closely related sequences. Numbers at nodes are levels of bootstrap support (%) based on Neighbor-joining (NJ) method of 1,000 resampled datasets. The bootstrap value below 60% is not shown. The scale bar indicates 0.01 nucleotide substitution per position. Isolate is marked.

**Characterization of isolate and phylogenetic analysis.** The isolated bacterium was Gram negative, rod shape and motile. The results of the biochemical tests are shown in Table 1. Biochemical characterization revealed that isolate POS-F84 might be similar to *P. putida* according to the description in Bergey's manual of Determinative Bacteriology (15). The 16S rDNA sequence of strain POS-F84 showed 99% and 98% similarity with the corresponding sequences of *P. putida* and *P. entomophila*, respectively. Multiple alignment and phylogentic analysis revealed the strain was closely related to *P. putida* (Fig. 2). However, with considering the close relation between *P. putida* and *P. entomophila* (70.2% of *P. entomophila* genes have orthologs in the *P. putida* genome), this difference of 1% was not enough for exact identification (23, 24). For this reason, we sequenced the ITS region to infer accurate discrimination of isolate POS-F84.The ITS nucleotide was analyzed with the BLAST program and showed 97% and 94% homology with the ITS of *P. entomophila* and *P. putida* strains, respectively. According to the created phylogenetic tree based on ITS sequences (Fig. 3), it was concluded that the strain POS-F84 was more closely related to *P. entomophila* than were *P. putida*. Therefore, this strain was recognized as *P. entomophila*.

**Table 1:** Biochemical properties of the POS-F84 isolate.

Characteristic	pOS-F84
Triple Sugar Iron Agar (TSIA)	Alkaline/Alkaline
Methyl red (MR)	−
Citrate	+
Voges-Proskauer (VP)	+
Indole production	−
Urea hydrolysis	−
Oxidase reaction	+
Catalaze	+
H S Production	−
Inositol fermentation	−
Mannitol fermentation	−
Sorbitol fermentation	−
Arginine dihydrolase	+
L-Rhamnose fermentation	−
Saccharate	−
α-Amylamine	−
Lysine Decarboxylase	−
L-Arabinose fermentation	+
O-Nitrophenyl-β-D-Galactopyranoside	−
Glucose	+
Gelatin hydrolysis	−
Lecithinase (egg yolk reaction)	−
Levan formation from sucrose	−
Ornithine Decarboxylase	−

Symbols: + and −, positive and negative for biochemical reactions.

**Fig. 4:**
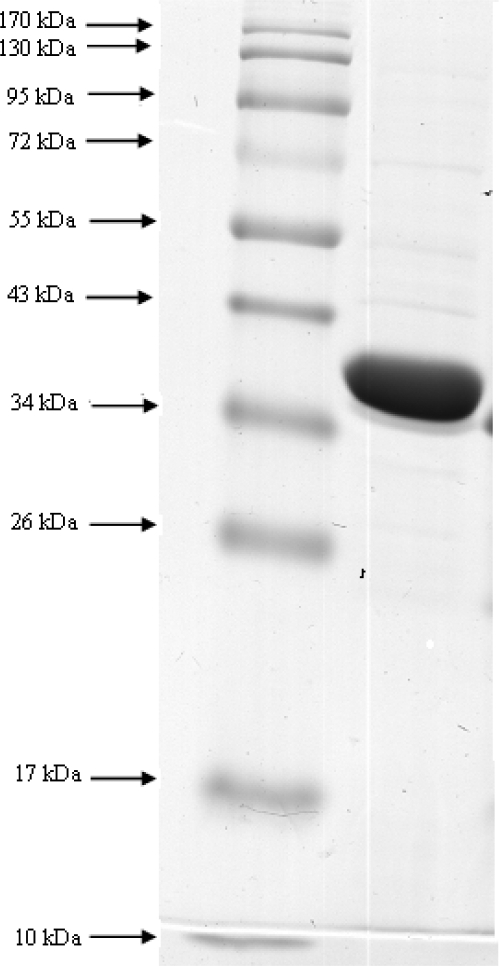
SDS-PAGE electrophoresis of ProDH. Lane 1: molecular standard markers lane 2: sample from sephadex G-200 column.

**Characterization of *P. entomophila* ProDH.** The ProDH with a single band of 40 kDa on native PAGE (data not shown) was purified to homogeneity from the *P. entomophila* with a yield of 8.5% and a purification factor of 37.3. SDS-PAGE gel analysis of purified target enzyme showed a single band with an estimated molecular mass of 40 kDa (Fig. 4). As the result, the purified ProDH was a monomeric protein. The substrate specificity of the ProDH reaction with different substrates was examined. L-proline (100%) was the most preferred substrates for ProDH reaction. The enzyme also showed weak activities towards L-Threonine, L-Alanine and Acetaldehyde.

**Fig. 5:**
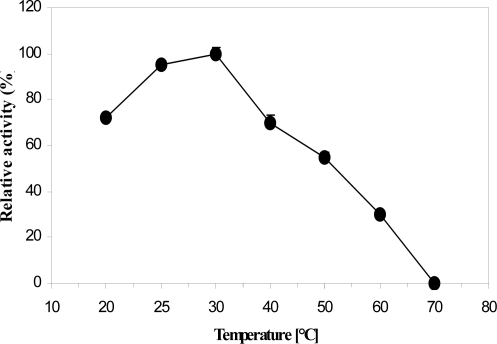
Influence of temperature on the activity of *P. entomophila* ProDH.

**Fig. 6:**
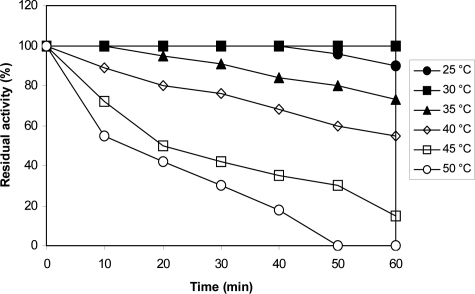
Influence of temperature on the stability of ProDH reaction from *P. entomophila*.

**Fig. 7:**
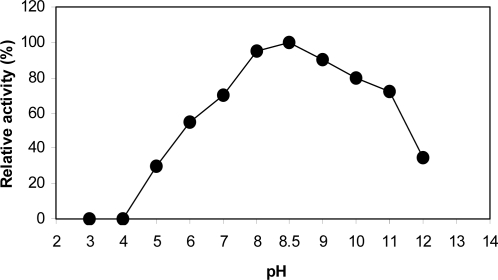
Influence of pH on the ProDH reaction of *P. entomophila*.

**Fig. 8:**
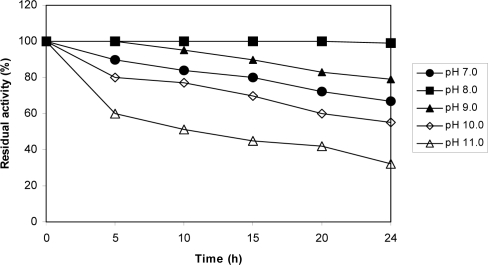
Influence of pH on the stability of ProDH reaction from *P. entomophila*. Enzyme sample were incubated in different buffers and aliquots were withdrawn at different time intervals for enzyme assay.

The following amino acids were inert for the ProDH reaction: D-proline, L-Hydroxyproline, L-Arginine, Aspartate, and Glycine. Moreover, chelating agents such as EDTA did not inhibit the enzyme. The *K*_m_ value of ProDH reaction for proline was also calculated to be 32 mM. The ProDH reaction exhibited its maximal activity at temperature range of 25 to 30°C, and its highest activity was achieved at 30°C (Fig. 5). As can be seen (Fig. 5), a sharp decrease in enzyme activity was observed above 30°C and was completely inactivated at 70°C. For examination of the temperature effect on enzyme stability, the residual activity of ProDH incubated at different temperatures (25–50°C) for a period of two hours was measured (Fig. 6). The ProDH was almost stable at temperatures between 25–30°C for two hours, but lost 22%, 35% and 45% of its initial activity after incubation for two hours at 35, 40 and 45°C, respectively. At 50°C, target ProDH was completely inactivated after 40 min. The effect of various pH values on the enzymatic reaction of ProDH was evaluated in the pH range from 3.0 to 12.0 at 30°C. ProDH had a good activity in the range of pH 7.0–9.0 with optimal pH at 8.5 (Fig. 7). The effect of pH on enzyme stability was also tested by the measurement of residual activity after incubation at different pH values for 24 hours. ProDH was highly stable between pH 8.0 and pH 9.0, while at pH 10, it only retained 50% of its original activity during the same period incubation (Fig. 8).

## DISCUSSION

The amino acid L-proline is catabolized into glutamate by the actions of two enzymes, ProDH and P5CDH (4). The bacterial enzymes participating in proline metabolism are potentially attractive models for studying the structure and function of human homologues and also specific determination of plasma L-proline in diagnostic kits and biosensors (5, 6). Considering the medical applications of enzymes catabolizing proline in diagnosis of inborn errors of proline metabolism, we attempted to isolate these target biocatalysts from soil-inhabiting organisms. At the end of screening program, only strain POS-F84 isolated from the river clay, was found to be capable to produce ProDH and selected for further studies. To identify the strain POS-F84, biochemical and genetic analysis was performed. Phylogentic analysis revealed that the strain was a newly isolated *P. entomophila*. SDS-PAGE gel analysis of purified target enzyme showed a single band with an estimated molecular mass of 40 kDa. The reported molecular mass for this enzyme varies from 40 kDa to 45 kDa. Therefore, the molecular weight of our target protein was in agreement with the available observations for ProDH enzyme. Similar results have been observed for *P. aeruginosa* (10) and *S. typhimurium* (9) ProDHs. The *P. entomophila* ProDH possessed the different kinetic characteristics from the other ProDH and had lower *K*_m_ value for proline than that reported so far. As it has been noted in the literatures, high *K*_m_ value of ProDHs for proline is one of the common features of proline metabolizing enzymes in bacteria (5). For example, *K*_m_ value for proline for the ProDH enzymes in *P. aeruginosa* (10) and *S. typhimurium* (4) has been reported 45 mM and 43 mM, respectively. Therefore, the higher affinity of *P. fluorescence* ProDH toward proline made this enzyme very attractive for use in biosensors and protein engineering studies.

In summary, a ProDH was purified and characterized from a *P. entomophila* strain isolated from a river clay sample. Collectively, ProDH from *P. entomophila* appears to be useful for application in L-proline biosensor. To best of our knowledge, there is no report about production of ProDH by *P. entomophila*.
